# Study Protocol for the PARTICIPATE Project: Co‐Creating Resources for Public Engagement in Maternity Care Clinical Audit and Learning

**DOI:** 10.1111/hex.70491

**Published:** 2025-11-14

**Authors:** Tamara Escañuela Sánchez, Indra San‐Lázaro Campillo, Joye McKernan, Paul Corcoran, Richard A. Greene, the PARTICIPATE Project Advisory Group

**Affiliations:** ^1^ Department of Obstetrics and Gynaecology, National Perinatal Epidemiology Centre (NPEC) University College Cork Cork Ireland

**Keywords:** co‐design, maternal health, maternity clinical audit, participatory research, patient and public involvement (PPI), user engagement

## Abstract

**Introduction:**

Public and patient involvement (PPI) is increasingly recognised as essential in health research, service development and clinical audit. However, while Irish national guidance encourages PPI in audit, there is limited evidence on how to implement it meaningfully in maternity care. The PARTICIPATE Project aims to address this gap by exploring awareness and barriers to PPI in maternity clinical audits in Ireland, co‐creating accessible resources to support engagement, and establishing sustainable PPI panels for ongoing collaboration with a national clinical audit centre.

**Methods:**

This mixed‐methods study is led by the National Perinatal Epidemiology Centre and informed by participatory principles and international models such as the ENGAGE Project. The study is structured into three phases. Phase 1 consists of a national online survey exploring public awareness, interest, and perceived barriers to engagement in maternity clinical audit. In Phase 2, co‐design sessions will be conducted with members of the public and service users to develop practical, user‐informed resources to support future PPI activities. Phase 3 involves the creation of PPI panels to contribute to audit and research activities within the audit centre. Data collection and analysis will follow ethical standards, with support from the PARTICIPATE Project Advisory Group.

**Conclusion:**

This project will generate practical guidance on how to embed PPI in maternity clinical audit in a feasible and meaningful way. The focus on co‐produced resources and long‐term engagement structures aims to support more inclusive clinical audit practices and reinforce public confidence in the maternity care system.

**Patient or Public Contribution:**

An advisory group, including individuals with lived experience and expertise in maternity care and PPI has informed the development of the study protocol and survey so far. Public and patient contributors will also co‐produce project resources and be invited to participate in long‐term advisory panels. All contributors will be acknowledged in project outputs and publications.

## 1 Introduction

The integration of public and patient involvement (PPI) represents an important paradigm shift in healthcare, moving from a paternalistic model to one that embraces shared decision‐making and collaborative improvement. Public and patient inclusion is considered a necessary aspect of health research, audit and service development [1]. Clinical audit is a tool used to systematically assess and review current healthcare practices against explicit criteria.

Integrating PPI knowledge into clinical audits addresses several key factors, including enhancing transparency and accountability, improving the quality and relevance of the audit, and supporting patient empowerment [2]. Clinical audits that actively involve PPI contributors are more likely to identify areas of healthcare that are most relevant to their experiences and priorities [3, 4]. When PPI is carried out in a meaningful and sustained way, this focus can lead to improvements that are meaningful for those who use the services, promoting a sense of ownership and shared responsibility for quality improvement [5]. However, poorly integrated approaches may fail to achieve these benefits. By incorporating patient feedback and insights, organisations can gain a deeper understanding of the barriers and facilitators to optimal care, leading to more targeted and effective audits [6].

While national guidance encourages PPI in clinical audit [7], little evidence exists on how to do this well, particularly in maternity care. Most of the available literature focuses on PPI involvement in research, and even in that regard, previous research has concluded that there was limited evidence about the extent of PPI involvement reporting in studies related to maternal health research [8]. Furthermore, multiple workforce barriers have been identified, including time or capacity issues, lack of skills and staff resistance [9, 10]. In addition, barriers and facilitators on the PPI side include perceived ability to contribute, lack of diversity, and accessibility of opportunities [9, 10, 11]. Addressing both workforce and participant‐related barriers is therefore essential for effective and sustainable PPI in audit processes.

Clinical audit involves sensitive reflection on care provision, and in maternity care, this is particularly complex given the emotional impact of pregnancy outcomes and issues of trust between families and providers. National audit programmes such as the National Perinatal Mortality Audit in Ireland and the National Maternity and Perinatal Audit in the UK explicitly recognise the sensitive nature of maternity outcomes and the importance of engaging service users in interpreting and sharing results [12, 13].

In the UK, national quality improvement programmes such as the Healthcare Quality Improvement Partnership (HQIP), the National Neonatal Audit Programme (NNAP), and Maternity Voices Partnerships (MNVPs) have developed structured approaches to PPI, demonstrating the value of systematic engagement strategies in maternity and neonatal care [14, 15, 16]. These initiatives are supported by long‐standing policy drivers that make PPI a formal expectation across quality improvement. In contrast, Ireland has only recently begun to embed PPI within healthcare policy (HSE, 2021 14), and maternity audit structures do not yet incorporate consistent or formalised approaches to public involvement. When it comes to PPI, maternity care poses especial challenges. Pregnancy and birth are highly emotional life events, and participation in audit or improvement discussions may evoke distress, especially for those who have experienced complications or bereavement. Women may also feel vulnerable in sharing critical views due to perceived power imbalances with healthcare. Historical controversies such as non‐consensual symphysiotomies and failures in the CervicalCheck screening programme in Ireland illustrate how lack of transparency in women's health has eroded public trust [17, 18]. Practical barriers such as childcare responsibilities, limited time, and capacity can further restrict involvement. This project therefore, builds on lessons from UK programmes but adapts them to the unique Irish context, where these challenges create both the need and opportunity for new, co‐produced strategies for audit engagement.

Improvements in maternity care are critical not only because of their potential to reduce mortality and morbidity, but also because of their impact on longer‐term maternal and infant health and public trust in healthcare services. Evidence consistently shows that outcomes in maternity care are not equally distributed. Women from ethnic minority backgrounds, women experiencing social disadvantage and migrant women are at increased risk of adverse maternal and perinatal outcomes (CITE MBRACE). Addressing these inequalities requires not only clinical improvements, but also approaches that ensure maternity audit processes reflect the voices and priorities of diverse service users. Maternity care involves complex experiences that cannot be fully captured through quantitative audit data alone. Qualitative approaches and PPI are therefore essential to ensure that audit processes reflect the lived realities and priorities of service users. This is why promoting PPI in the design, implementation and interpretation of maternity clinical audits is essential to ensure that these processes are not only clinically robust, but also respond to the people they aim to serve.

Exploring alternative methods to involve patients and the public in clinical audit is necessary to address these barriers, and to do so, understanding their experiences of participating in clinical audit is essential.

The study phases aim to answer the following research questions:

This study aims to answer the following questions:

1. What is the level of public awareness of, and what are the perceived barriers to, involvement in maternity clinical audits in Ireland? (Phase 1)
2. How can evidence generated from the national survey be used to inform and strengthen PPI policies and practices in maternity clinical audit? (Phase 1)
3. What formats, content, and processes are considered acceptable, relevant, and feasible by PPI contributors for resources that support engagement in maternity audit? (Phase 2)
4. What models of patient and public panels are feasible and sustainable in the context of maternity audit in Ireland, and what levels of involvement are acceptable to patient and public contributors over time? (Phase 3)

This project will contribute to the limited evidence base on how to implement meaningful PPI in clinical audit, particularly in perinatal care. By exploring public awareness and perceptions through a national online survey, we will generate new knowledge into current levels of understanding and interest in perinatal care audit engagement in Ireland. The project will also aim to co‐produce practical, user‐friendly resources to support PPI engagement in perinatal care clinical audit that will be available to other organisations or research teams. The findings of this project will inform a set of outputs that will provide a replicable model and set of tools that may inform future audit processes.

Amendments made to this protocol will be acknowledged in any publications following this study.

## 2 Methods

The PARTICIPATE project design has been informed by the participatory model developed in the ENGAGE project conducted at the CRE Stillbirth Australia [19, 20], which aimed to promote partnerships between bereaved parents and researchers in the co‐design and prioritisation of stillbirth research. The PARTICIPATE project has adapted key elements of the ENGAGE project methodology to the context of clinical audit. Adaptations have been informed both by a review of the ENGAGE project's published materials and through direct consultation with the original project team, further, a member of the Australian research team has been invited to participate in the PARTICIPATE project advisory group. From ENGANGE, the PARTICIPATE project has adopted several key elements: the use of an advisory group including both professionals and people with lived experience to guide all stages of the project; an initial exploratory survey to assess awareness but also to recruit participants for the next phases of the project; participatory co‐design workshops to develop outputs that are acceptable and relevant for the end‐users; and interactive feedback cycles to refine resources and processes.

To support this project, a Data Advisory Group with members from different national and international organisations has been established. The purpose of this group is to bring expertise in different fields and produce continuous feedback alongside the development of the project.

### 2.1 Project Design

This project is based on a series of mixed methods studies and activities grounded in the need to capture a wide range of experiences both quantitatively and qualitatively. Quantitative responses from the survey will serve to provide a general overview of awareness, interest, and potential barriers from a wider sample, whereas qualitative data from the open‐ended survey questions and the co‐design sessions will offer richer and deeper insights into lived experiences, preferences, and contextual nuances.

This project is adopting a participatory design to ensure that the development of future materials is informed by those with lived experiences in perinatal care. This approach aligns with established frameworks for PPI, which highlight the importance of co‐production in producing interventions that are not only relevant and acceptable to intended users, but also more likely to be sustainable in time [8].

### 2.2 Project Phases

The project is divided into three main phases. A summary of the aims, methods, and outcomes of each phase can be seen in Table 1. PPI contributors will not receive payment for their involvement in any of the phases due to a lack of specific funding.

**Table 1:** Participate project overview.

Phase	Aim	Methods	Output	Type of activity
Phase 1	Understand awareness and perceptions	National online survey (mixed methods)	Baseline data on knowledge, barriers, and interest in PPI in maternity audit	Research
Phase 2	Co‐create resources with service users	Working groups, co‐design sessions	Practical, user‐informed materials to support PPI in perinatal care clinical audit	PPI
Phase 3	Build sustainable PPI structures	Creation of public/patient advisory panels	Long‐term involvement in the centre's activities, including governance structure membership	PPI

#### 2.2.1 Phase 1. Survey Study

Phase one will consist of a national online mixed methods survey that will aim to explore awareness and preferences regarding PPI in clinical audit in Ireland. The findings from this survey will inform the rest of the phases of this project. The survey has been developed in collaboration with the PARTICIPATE advisory group, which includes representatives from a range of stakeholder organisations, such as national bodies, patient advocacy groups, and charities. The group also includes public and patient contributors who have been actively involved in the audit centre's activity to date. The survey will be piloted by a colleague who has recently been pregnant and is not part of this study team. While resource and timeline constraints limited broader piloting and we acknowledge this as a limitation of this study, input was also incorporated from the advisory group and research team members, who represent a range of ethnic and professional backgrounds.

Eligibility will include people aged 18 years or older who have either: (a) used maternity services in Ireland within the last 5 years; or (b) supported a partner, friend, or family member through maternity care. ‘Experience of maternity care’ thus refers to either direct use of services or supporting roles. Individuals without such experience will not be eligible to participate.

The survey will be distributed using our social media channels, as well as the channels of our collaborating organisations, primarily via a convenience sample. Paper copies will not be made available in the first stage of recruitment, which may have limited participation among individuals with reduced internet access. To increase diversity, purposive outreach will also be undertaken in partnership with organisations representing underrepresented groups (e.g., migrant, Traveller, or disabled women), we will discuss with the different community representatives if other methods of completing the survey (e.g., paper copies, guided reading, translation) are necessary. An online survey will be designed using REDCap with input and feedback from the data advisory group. The survey contains approximately 20 questions and takes approximately 10 min to complete. The survey includes both multiple‐choice and open‐ended questions. exploring the following areas:

- Demographic information, including age, sex, ethnic group, disability status, education level, employment status, which maternity service they engaged with, and experience with maternity care (including no major complications, bereavement, maternal morbidity, termination of pregnancy, homebirth, multiple pregnancy, etc).
- Knowledge and awareness regarding clinical audit.
- What type of activities have people engaged in, if any.
- Barriers to engagement in PPI activities.
- Strategies for engagement in PPI activities.
- Resources needed and preferred format.
- Interest in participation in further activities.

Although some survey items are phrased in general terms, eligibility criteria ensure that responses reflect experiences of maternity care. Broader items on strategies for engagement were included intentionally to inform subsequent co‐production and panel development phases, while analysis and reporting will remain focused on the maternity care context.

This is an exploratory, national online survey, designed to maximise reach through wide distribution. Based on previous national surveys conducted by NPEC, approximately 150−200 responses are anticipated.

Responses will be exported from REDCap and imported into SPSS (IBM SPSS Statistics for Windows, Version 28.0. Armonk, NY: IBM Corp) for analysis. The analysis will include descriptive statistics and cross‐sectional tables. Open‐ended questions will be imported into NVIVO and analysed using reflexive thematic analysis as proposed by Braun and Clarke [21].

No identifying information will be required to complete the survey. If participants choose to provide contact details for future involvement, these will be collected in a separate optional form, stored securely, and not linked to survey responses. All data will be stored on password‐protected servers with access limited to the research team. Incomplete survey responses (less than 80% completion) will be excluded from analysis.

#### 2.2.2 Phase 2. Co‐Production of Resources

The aim of this phase is to co‐create resources with patients and members of the public that are accessible, relevant, and useful to a wide range of people. The development of these resources will be guided by the survey findings and further shaped through patient working groups, which will explore their format, content, and distribution strategies. We expect to create the patient working groups through the expressions of interest indicated in the survey.

The aim of these resources is to support greater awareness, understanding, and confidence in contributing to PPI within maternity audits. The primary audience will be patients and members of the public with experience of maternity care, although practitioners and audit teams may also use the resources to support or facilitate involvement. The decision on whether separate resources are required for different audiences will be guided by the co‐design process, ensuring that outputs are tailored to the needs and preferences of those who will use them.

Participants who consent to further involvement in their responses to the survey will be purposively selected to ensure a diversity of perspectives across demographic backgrounds, lived experiences, and levels of previous involvement. Sessions will be conducted in person or online, depending on participant's preferences. Working groups will be facilitated using inclusive and participatory approaches, such as guided discussions, visual online tools, and collaborative decision‐making methods such as Padlet or Mentimeter.

Input from the working sessions will be documented through anonymised recordings and transcriptions. Audio from the groups will be recorded with permission. The discussions from the working sessions will be used to draft and refine the resources produced. Participants will be offered the opportunity to review and comment on all the different versions of the resources. All contributors will be acknowledged appropriately. Phase 2 is categorised as a PPI activity, we anticipate involving approximately 10−15 participants across several working groups, purposively selected from survey respondents who indicated willingness to be contacted. Selection will be based on demographic information collected in the survey to ensure representation across key groups.

#### 2.2.3 Phase 3. Panel Creation

The aim of this phase is to create panels of PPIs with an interest in being involved in the audit centre's audit and research activities. The survey administered in the first phase of this study will explore participant's experiences with maternity services. Participants will be identified from those who complete the survey in Phase 2 and indicate interest in further involvement. As part of the survey, respondents will be asked whether they would like to be contacted about future opportunities to contribute to the project in a PPI capacity.

Interested individuals will be invited to a structured onboarding process, in accordance with the centre's existing PPI policy. This policy includes meeting the director of our centre to explore questions and expectations, once this process is completed, the PPI has an induction with the rest of the centre's team and previous.

The PPI panels will not function as research participants but as co‐investigators or advisory contributors. Their insights will be integrated into audit planning, interpretation of findings, dissemination materials design, and dissemination strategies.

Support and recognition for their contribution will be provided in alignment with national guidance [22]. Participation in the panels and all the subsequent activities will be entirely voluntary. The centre will maintain a flexible approach to PPI involvement, allowing PPIs to engage on a topic‐specific basis or in a more ongoing role, depending on their interest and capacity.

To ensure long‐term sustainability of the PPI panels, several considerations need to be made. To do so, we will review our PPI policy with input from the advisory group and PPIs that engage in the project. This will include revising aspects such as funding and resources, staff support, reviewing our structures and processes, considerations regarding recruitment of new members, expectations about commitments and outputs, training and capacity building. Regular feedback will be gathered to inform continuous improvement. PPI contributions will be integrated into the centre's governance and strategic plans.

Panel size may vary depending on the type of maternity care experience represented. For example, more common experiences (such as routine maternity care) may be represented by larger panels, while less common but equally important experiences (such as stillbirth or severe maternal morbidity) may be represented by smaller panels. This flexible approach will allow us to ensure diversity of perspectives while maintaining feasible and sustainable structures.

#### 2.2.4 Evaluation of Phases 2 and 3

Although Phases 2 and 3 are categorised as PPI activities rather than primary research, they are underpinned by exploratory questions and will include formative evaluation. Phase 2 will assess the feasibility and acceptability of co‐producing resources with service users, focusing on preferred formats, content, and processes. Phase 3 will examine the feasibility and sustainability of establishing PPI panels in the maternity audit context, including the acceptability of different levels of involvement and models of participation. Sustainability will be defined as the capacity of the panels to be maintained over time, reflected in recruitment and retention of members, diversity of representation, ongoing contributor engagement, and integration into audit governance structures. Evaluation across both phases will involve documenting recruitment and attendance, monitoring membership diversity, and collecting structured feedback from participants and advisory group reflections to provide early evidence on the feasibility, acceptability, and sustainability of PPI in maternity audit.

## 3 Ethics and Dissemination

The University College Cork (UCC) Code of Research Conduct and the General Data Protection Regulations (GDPR) procedures are being followed for all research activities. Ethical approval for Phase 1 has been granted by the Social Research Ethics Committee at UCC (ECM 4 (cc) 15/07/2025) & ECM 3 (j) 12/08/2025).

Regarding phase 1, participants will be provided with information sheets and request to provide informed consent when opening the online survey. Participants will be informed that if they are able to withdraw from the study at any time before submitting the survey, but that once submitted, this possibility will no longer be available. Responses that do not reach 80% of completion will be treated as withdrawals and excluded from analysis.

Phases 2 and 3 of the PARTICIPATE study involve Patient and Public Involvement (PPI) activities, rather than primary research. These phases are designed to engage participants in co‐design and advisory processes to shape the study, not to collect or analyse personal research data. As such, they do not fall under the remit of formal ethical review.

Participants will have already provided informed consent in Phase 1 to be contacted about opportunities to take part in PPI activities. Participants will be informed that all engagement in Phases 2 and 3 will remain entirely voluntary, and participants can withdraw at any time.

Regarding phase 2, participants will receive information materials about the co‐production meetings. They will be informed that participation is voluntary and that they can withdraw at any time with no consequences to their care and without giving any reasons. Participants will be informed that meetings will be recorded. Written informed consent will be obtained to participate in the meetings. Participants will be informed that all data will be stored anonymously.

Regarding phase 3, ongoing support will be provided to PPI contributors that decide to participate in the centre's panels and activities. Their time and capacity will be always considered when inviting them to participate in activities. PPI contributors will be reassured that their participation continues to be voluntary and that they can accept and decline any of the activities proposed with no consequences.

Participants are not expected to face any physical risks as part of their involvement in the project. However, discussions related to maternity care may sometimes evoke distress or bring up difficult personal experiences. To safeguard contributors, structured support will be available if required. Members from the clinical team will be available to discuss any potential issues or concerns that PPI contributors might have, and signposting to relevant services will be provided if necessary if contributors experience distress or trauma during the process.

Findings and resources produced through the project PARTICIPATE will be disseminated through different channels. Academic dissemination will include submission of articles to peer‐reviewed journals and potential presentations at national or international conferences. The project will also be disseminated through the centre's website and social media platforms, both to encourage recruitment and to share findings and resources produced. The advisory group and all PPI collaborators will be acknowledged in dissemination activities as appropriate. The resources co‐developed in Phase 2 will be made publicly available to support broader adoption of PPI practices in clinical audit.

## 4 PPI

An advisory group including individuals with expertise in PPI, clinical audit, and maternity services, as well as people with lived experiences, has been established to provide feedback and guidance throughout the project. Members of this group will contribute to refining and reviewing the project protocol and plan, survey content, advising on recruitment strategies, and sharing recruitment materials, as well as providing feedback all throughout the project. The advisory group will be acknowledged in all publications resulting from this project and on the project's website.

During the study, a different group of patient and public contributors will also be involved in co‐producing resources (Phase 3) and will be invited to participate in PPI panels for future activities (Phase 4). Their contribution will be valuable to enhance the shape of our audit and research activities, as well as to ensure that our findings are translated into user‐friendly resources.

## 5 Timeline

It is anticipated that the duration of this study is 2 years.

## 6 Study Status

At the moment, the advisory group has had its first three meetings, which focused on designing the survey and the dissemination strategy. Ethical approval was obtained on the 23rd of July 2025, so the survey has been disseminated through different social media channels. Phase 1 of this study is expected to be completed by the end of 2025.

## 7 Discussion

In summary, following this process will hopefully allow us to identify what are the barriers and facilitators to encourage PPI contribution into clinical audit, develop resources that can be used with other organisations, and create patient panels to enhance our own activity. Availing of an advisory panel with wide expertise will ensure that the feasibility of the project is always considered. In doing so, the project seeks to contribute to a more structured but adaptable, inclusive, and patient‐centred approach to clinical audit.

We expect that the co‐produced resources will support future sustainability of the project by building confidence amongst the public to participate in PPI activities, but also amongst other organisations to confidently incorporate patients and members of the public into their activities. While the focus of this project is on perinatal care services, we hope that the methods, tools and co‐production developed may be transferable to other areas of healthcare audit.

### 7.1 Strengths and Potential Challenges of This Study

The main strength of the proposed project is that it addresses a clear gap in the literature by utilising a participatory, co‐designed approach. This approach, which includes an advisory group with relevant stakeholders and members of the public and the plans to co‐design resources with other patients, will enhance the rigour and relevance of the outputs produced.

As mentioned above, most PPI literature focuses on research, the aim of this project is to expand the knowledge of PPI in a the clinical audit context, especially related to perinatal care. The findings and tools developed will be relevant across all Irish maternity system and potentially applicable to other contexts.

Lastly, the use of mixed methods allows the study to capture both broad patterns and deeper insights. The quantitative survey will enable engagement with a larger and more diverse sample, helping to identify general trends in awareness, barriers, and interest in PPI. Meanwhile, the inclusion of open‐ended survey questions and co‐production workshops with patients and members of the public will allow for more detailed exploration of key themes. Given the wide variability in perinatal care experiences, this combined approach is particularly appropriate to ensure both representativeness and depth of understanding.

We identify a number of potential challenges, including difficulties in recruiting participants from underrepresented groups, sustaining engagement over time, and supporting an ambitious project with limited resources for PPI activities. Additional limitations include piloting the survey with only one colleague, which restricted early testing but was mitigated through advisory group input; offering the survey only online, which may reduce accessibility for those without digital access; and the lack of payment for contributors due to limited funding, which may affect participation, although recognition and acknowledgement will be provided. Some survey items were also framed in general terms, which may appear broader than the specific focus on maternity PPI, but this was intentional to inform subsequent co‐production and panel phases. To address this, the advisory group includes multiple levels of expertise and members who can support recruitment of specific groups (e.g., disabilities or ethnic minorities). The project will also maintain a flexible nature where all collaborators and PPI will be able to choose their level of involvement.

## Author Contributions

T.E.S. contributed to the study conceptualization, methodology, project administration, software, and writing – original draft preparation. I.L.C. and J.M.c.K. contributed to the study conceptualization, methodology, and writing – review and editing. P.C. and R.G. contributed by providing supervision and writing – review and editing. Members of the PARTICIPATE advisory group contributed to this study by supporting conceptualization, methodology, and writing – review and editing. All authors approved the final version of the manuscript.

## Ethics Statement

Ethical approval for the study has been granted for phase 1 (survey study) by the Clinical Research Ethics Committee at UCC (ECM 4 (cc) 15/07/2025 & ECM 3 (j) 12/08/2025).

## Conflicts of Interest

The authors declare no conflicts of interest.

## Data Availability

Data sharing is not applicable to this article as no datasets were generated or analysed during the current study.
